# Interpretable machine learning for in-hospital mortality prediction in ICU patients with traumatic brain injury

**DOI:** 10.3389/fneur.2026.1815307

**Published:** 2026-04-23

**Authors:** Ning Liu, Congcong Li

**Affiliations:** Affiliated Taihe Hospital of Wannan Medical College, Fuyang, China

**Keywords:** in-hospital mortality, interpretability, machine learning, MIMIC-IV, SHAP, traumatic brain injury

## Abstract

**Background:**

TBI is associated with high ICU mortality, yet traditional prognostic scores often lack accuracy due to linear assumptions. This study aimed to develop an interpretable machine learning model to predict in-hospital mortality in TBI patients, combining high predictive performance with clinical transparency.

**Methods:**

This retrospective analysis utilized TBI clinical records (2008–2019) retrieved from the MIMIC-IV database. We collected comprehensive baseline data including demographics, comorbidities, vital signs, laboratory parameters, disease severity scores, and therapeutic interventions. To identify the most robust predictors, we employed a rigorous intersectional feature selection strategy combining Univariate Logistic Regression (*p* < 0.05), LASSO regression, and the Boruta algorithm. Seven supervised ML algorithms (Logistic Regression, Decision Tree, Random Forest, XGBoost, LightGBM, SVM, and ANN) were developed and compared. Predictive performance was benchmarked using AUROC, Brier score, and DCA. Additionally, SHAP were implemented to enhance the interpretability of the final model’s decision-making process.

**Results:**

Among the 2,691 included TBI patients, 1,716 (63.8%) were male, and the median age was 66 (IQR 48–81) years. The primary outcome, in-hospital mortality, occurred in 316 patients (11.7%). A final predictive set of 12 variables was identified: demographic and clinical metrics (age, GCS, temperature), lab results (anion gap, glucose, PT, RDW, WBC, urea nitrogen), and key clinical interventions or conditions (acidosis, mannitol, and sedatives). Among the seven algorithms, the XGBoost model achieved the best performance with the highest AUROC of 0.873 (95% CI: 0.842–0.906) and superior calibration (Brier score = 0.078). SHAP analysis identified GCS, age, anion gap, and glucose as the top mortality drivers, and revealed critical non-linear relationships, such as a U-shaped association between body temperature and mortality risk.

**Conclusion:**

We successfully developed and validated an interpretable XGBoost-based model that demonstrates robust discriminative capacity, excellent calibration, and high sensitivity for predicting in-hospital mortality in ICU patients with TBI. By integrating SHAP analysis, the model provides intuitive explanations for both population-level risk drivers and individual predictions, effectively bridging the gap between predictive accuracy and clinical interpretability to facilitate personalized decision-making in the ICU.

## Introduction

Global health landscapes are significantly impacted by TBI, which represents a leading etiology of fatal outcomes and chronic neurological deficits. Globally, an estimated 69 million individuals suffer from TBI annually, placing an immense burden on healthcare systems (1, 2). The condition is particularly severe in the ICU setting, where mortality rates for severe TBI remain alarmingly high, ranging from 30 to 50% despite advances in neurocritical care (2, 3). The economic impact is equally profound; in the United States alone, the total annual cost of TBI, including direct medical expenses and lost productivity, has been estimated to exceed $76 billion (4, 5). Given the high incidence, poor prognosis, and substantial resource consumption, TBI represents a critical challenge that demands urgent attention.

In this high-risk population, early and accurate prediction of in-hospital mortality is clinically vital. Rapid identification of patients at the highest risk of death allows clinicians to optimize resource allocation and tailor aggressive therapeutic interventions promptly (6). Furthermore, reliable prognostic assessment is essential for facilitating shared decision-making with families and establishing realistic goals of care (7). Consequently, developing effective predictive tools is not just a technical objective but a fundamental requirement to improve management strategies and outcomes for ICU patients with TBI.

Currently, the prognostication of TBI in the ICU primarily relies on traditional scoring systems and regression-based models. While general severity scores like APACHE II are widely used in ICUs, they often lack specificity for neurotrauma patients (8). Consequently, TBI-specific models, notably the IMPACT and CRASH models, have been established as the clinical gold standards (6, 9). However, these tools are predominantly based on logistic regression, which assumes linear relationships between risk factors and outcomes. This linear assumption is often an oversimplification that fails to capture the complex, non-linear interactions and high-dimensional physiological patterns inherent in continuous ICU monitoring data, potentially limiting their predictive accuracy in individual patients.

Unlike conventional linear models, ML algorithms like LightGBM and Random Forest excel at interpreting massive, multi-dimensional ICU datasets through their capacity for non-linear modeling (10). Despite their superior predictive power, a critical barrier hinders the clinical translation of these advanced models: their “black-box” nature. Most high-performing ML models lack transparency, offering no insight into the logic behind a specific prediction. In the high-stakes environment of the ICU, predictive accuracy alone is insufficient; clinicians require interpretability to understand the underlying reasoning, validate the model against pathophysiological knowledge, and trust the results for life-and-death decision-making (10, 11). Consequently, there is an urgent need for interpretable ML frameworks that combine high predictive performance with the transparency required for clinical adoption.

## Methods

### Data source

This retrospective study utilized TBI clinical records (2008–2019) from the MIMIC-IV database (v2.0). We identified adult subjects (≥18 years) with a primary diagnosis of traumatic brain injury or craniocerebral trauma at ICU admission.

### Study population and eligibility criteria

**Figure 1:**
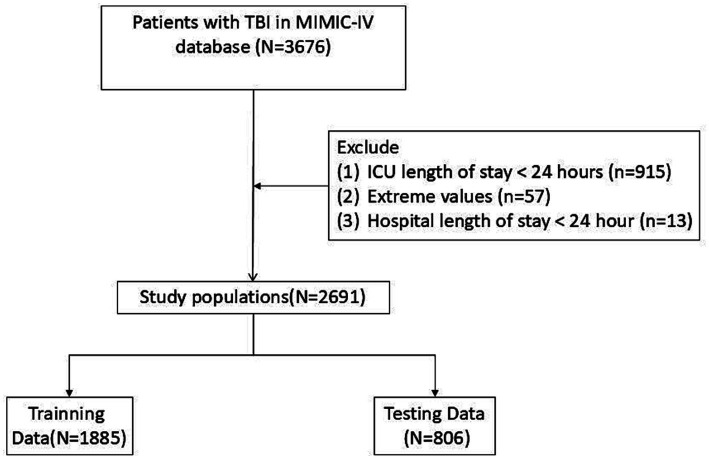
The flow chart of the participants selection.

Eligibility required age ≥18 and a confirmed TBI diagnosis, with the analysis focused solely on initial ICU encounters. To ensure data integrity, we excluded patients with abbreviated ICU stays (<24 h), incomplete mortality records, or extreme physiological outliers (Figure 1).

### Ethics approval

Data access from the MIMIC-IV repository was authorized by the Institutional Review Boards of Beth Israel Deaconess Medical Center and Massachusetts Institute of Technology. Given the retrospective design and use of public data, the requirement for informed consent and specific ethical approval was waived. This study adhered to the Declaration of Helsinki and all applicable institutional regulations.

### Data collection

Clinical data including demographics, comorbidities, interventions, severity scores, laboratory results, and vital signs were extracted from the MIMIC-IV database. For physiological variables, laboratory results, and the Glasgow Coma Scale (GCS) score, the initial recorded value within the first 24 h of ICU admission was utilized. Additionally, acute clinical conditions identified throughout the ICU stay, such as acidosis, sepsis, and acute kidney injury (AKI), were incorporated to capture the comprehensive clinical profile and disease severity of TBI patients.

Demographics included age (years), weight (kg), sex (male/female), and marital status (married, divorced, or other). Baseline comorbidities and acute conditions were systematically recorded, including: hypertension, diabetes mellitus, heart failure (HF), coronary heart disease (CHD), acute kidney injury (AKI), chronic kidney disease (CKD), malignancy (cancer), stroke, epilepsy, pneumonia, sepsis, acidosis, and delirium. Therapeutic interventions and pharmacological support included neurosurgical procedures, tracheostomy, mechanical ventilation, and platelet transfusion, alongside the use of vasopressors, sedatives, anticoagulants, antiplatelets, diuretics, beta-blockers, mannitol, thiamine, vitamin K, and insulin. Laboratory parameters were analyzed using standard clinical units, encompassing: white blood cell (WBC) count (×109/L), red blood cell (RBC) count (×1,012/L), platelet count (×109/L), red blood cell distribution width (RDW, %), serum electrolytes (sodium, potassium, total calcium, and chloride, all in mmol/L), blood glucose (mg/dL), anion gap (mmol/L), blood urea nitrogen (BUN, mg/dL), and serum creatinine (mg/dL). Coagulation profiles, including prothrombin time (PT, seconds) and international normalized ratio (INR), were also collected. Continuous vital signs included mean blood pressure (MBP, mmHg), respiratory rate (breaths/min), oxygen saturation (SpO2, %), and body temperature (T, °C).

The Glasgow Coma Scale (GCS) score, recorded upon ICU admission (initial value within 24 h), was included as a primary prognostic variable. The GCS is a standardized system for assessing the level of consciousness in patients with acute brain injury, evaluating motor, verbal, and eye-opening responses (12).

### Outcome

The main endpoint of this research was the occurrence of death during the initial hospitalization, termed in-hospital mortality. Patients who survived to hospital discharge or were transferred to another acute care facility were classified as survivors. The vital status was determined based on the hospital discharge records available in the MIMIC-IV database.

### Construction and performances assessment of the machine learning models

Given that this study involves a binary outcome, the sample size was determined based on the principle of having at least 10 events per variable (EPV) to ensure model stability and minimize overfitting.

A total of 2,691 patients were included (Figure 1). To ensure the integrity of model evaluation and prevent information leakage, the cohort was first stratified by in-hospital mortality and randomly divided into a training set (n = 1,885, 70%) and a validation set (n = 806, 30%). Subsequently, missing data were handled using Multiple Imputation by Chained Equations (MICE).

To identify the most robust predictors within the training cohort, we employed a tripartite feature selection strategy. Initial screening was conducted via univariate logistic regression, filtering candidates with a significance threshold of *p* < 0.05. Second, the Least Absolute Shrinkage and Selection Operator (LASSO) was utilized alongside 10-fold cross-validation to penalize redundant variables and mitigate multicollinearity by retaining only non-zero coefficients (13). Third, the Boruta wrapper algorithm which leverages random forest importance scores to contrast authentic variables against randomized shadow counterparts based on *Z*-values was implemented (14). To enhance predictive stability and curb the rate of false discoveries, the definitive variable set was restricted to the consensus of all three selection methodologies.

Seven machine learning algorithms were employed to construct predictive models using the selected features: Logistic Regression (LR), Decision Tree (DT), Random Forest (RF), eXtreme Gradient Boosting (XGBoost), Light Gradient Boosting Machine (LightGBM), Support Vector Machine (SVM), and Artificial Neural Network (ANN).

To refine model performance and curb the risk of over-parameterization, we implemented hyperparameter tuning via a grid search strategy integrated with 5-fold cross-validation (15). This systematic search prioritized the parameter configurations that yielded the maximum area under the receiver operating characteristic curve (AUROC) within the training cohort. We executed this optimization protocol independently across all five multiple-imputation datasets, ensuring that the final predictive models were developed using the most robust hyperparameter settings.

Model performance was rigorously evaluated across three dimensions: discrimination, calibration, and clinical utility. Discriminative capacity was quantified using the area under the receiver operating characteristic curve (AUROC). To assess the reliability of predicted probabilities, calibration was examined through calibration plots and the Brier score, measuring the congruence between estimated risks and actual outcomes. The practical value of each model was appraised via Decision Curve Analysis (DCA) to determine net benefits across a range of threshold probabilities. Furthermore, comprehensive diagnostic metrics including sensitivity, specificity, positive predictive value (PPV), negative predictive value (NPV), overall accuracy, and the F1-score were derived from the confusion matrix. The definitive model was prioritized based on achieving the peak AUROC in the validation cohort, alongside robust calibration and clinical utility profiles.

### Model interpretability

The inherent opacity of high-performing machine learning models often impedes their clinical utility, as the decision-making logic remains inaccessible to practitioners. To bridge the gap between algorithmic complexity and clinical interpretability, we employed SHAP (15–17). This technique, rooted in cooperative game theory, demystifies the decision-making process by assigning an importance value to each variable, thereby quantifying its specific contribution to the individual risk assessment. Global interpretability was visualized through: (1) summary bar plots ranking features by mean absolute SHAP values; (2) beeswarm plots displaying SHAP value distributions colored by feature magnitude to reveal directional effects; and (3) dependence plots illustrating non-linear relationships between feature values and mortality risk. Local interpretability was achieved through waterfall and force plots, which decompose individual risk scores into feature-specific contributions.

### Statistical analysis

Continuous variables were described as median (IQR) and compared via the Mann–Whitney U test, while categorical variables were presented as frequency (%) and analyzed using the chi-square test. Statistical significance was defined as a two-sided *p* < 0.05. Analyses were performed using R version 4.5.1.

## Results

A total of 2,691 ICU patients with TBI were included in this study, among whom 316 (11.7%) experienced in-hospital mortality. The cohort comprised 1,716 (63.8%) male patients, with a median age of 66 years (IQR: 48–81).

Compared with survivors, non-survivors were significantly older, had lower initial Glasgow Coma Scale (GCS) scores, and exhibited more pronounced abnormalities in laboratory parameters including renal function (creatinine, urea nitrogen), coagulation (PT, INR), glucose metabolism, and inflammatory or hematological markers (WBC, RDW, RBC, platelet count, anion gap) (*p* < 0.05). Significant differences were also observed in the prevalence of AKI, diabetes, heart failure, chronic renal disease, stroke, pneumonia, sepsis, and acidosis, as well as in the use of mechanical ventilation, sedatives, vasopressors, mannitol, diuretics, vitamin K, and platelet transfusion (*p* < 0.05). Conversely, no significant differences were found between groups regarding coronary heart disease (CHD), epilepsy, hypertension, cancer, delirium, neurosurgical surgery, or anticoagulant use (*p* > 0.05) (Table 1).

**Table 1:** Basic characteristics of participants.

Variables	Total (*n* = 2,691)	Survival (*n* = 2,375)	In-hospital (*n* = 316)	*p*-value
AKI (%)				<0.001
No	871 (32.37)	834 (35.12)	37 (11.71)	
Yes	1820 (67.63)	1,541 (64.88)	279 (88.29)	
CHD (%)				0.33
No	2,285 (84.91)	2023 (85.18)	262 (82.91)	
Yes	406 (15.09)	352 (14.82)	54 (17.09)	
Epilepsy (%)				0.711
No	2,496 (92.75)	2,205 (92.84)	291 (92.09)	
Yes	195 (7.25)	170 (7.16)	25 (7.91)	
Hypertension (%)				0.659
No	1,585 (58.90)	1,403 (59.07)	182 (57.59)	
Yes	1,106 (41.10)	972 (40.93)	134 (42.41)	
Diabetes (%)				<0.001
No	2,207 (82.01)	1975 (83.16)	232 (73.42)	
Yes	484 (17.99)	400 (16.84)	84 (26.58)	
HF (%)				<0.001
No	2,408 (89.48)	2,149 (90.48)	259 (81.96)	
Yes	283 (10.52)	226 (9.52)	57 (18.04)	
Cancer (%)				0.184
No	2,451 (91.08)	2,170 (91.37)	281 (88.92)	
Yes	240 (8.92)	205 (8.63)	35 (11.08)	
Chronic renal (%)				<0.001
No	2,454 (91.19)	2,188 (92.13)	266 (84.18)	
Yes	237 (8.81)	187 (7.87)	50 (15.82)	
Stroke (%)				0.006
No	2,519 (93.61)	2,235 (94.11)	284 (89.87)	
Yes	172 (6.39)	140 (5.89)	32 (10.13)	
Pneumonia (%)				0.002
No	2,119 (78.74)	1892 (79.66)	227 (71.84)	
Yes	572 (21.26)	483 (20.34)	89 (28.16)	
Sepsis (%)				<0.001
No	1,499 (55.70)	1,381 (58.15)	118 (37.34)	
Yes	1,192 (44.30)	994 (41.85)	198 (62.66)	
Acidosis (%)				<0.001
No	2,447 (90.93)	2,200 (92.63)	247 (78.16)	
Yes	244 (9.07)	175 (7.37)	69 (21.84)	
Delirium (%)				1
No	1,656 (61.54)	1,462 (61.56)	194 (61.39)	
Yes	1,035 (38.46)	913 (38.44)	122 (38.61)	
Gender (%)				0.002
Male	1716 (63.77)	1,540 (64.84)	176 (55.70)	
Female	975 (36.23)	835 (35.16)	140 (44.30)	
Marital status (%)				0.008
Married	963 (35.79)	872 (36.72)	91 (28.80)	
Divorced	140 (5.20)	127 (5.35)	13 (4.11)	
Other	1,588 (59.01)	1,376 (57.94)	212 (67.09)	
Mechanical ventilation (%)				0.002
No	2,233 (82.98)	1991 (83.83)	242 (76.58)	
Yes	458 (17.02)	384 (16.17)	74 (23.42)	
Neurosurgical surgery (%)				0.06
No	1938 (72.02)	1725 (72.63)	213 (67.41)	
Yes	753 (27.98)	650 (27.37)	103 (32.59)	
Tracheostomy (%)				0.193
No	1,476 (54.85)	1,314 (55.33)	162 (51.27)	
Yes	1,215 (45.15)	1,061 (44.67)	154 (48.73)	
Betablocker (%)				0.121
No	1844 (68.52)	1,640 (69.05)	204 (64.56)	
Yes	847 (31.48)	735 (30.95)	112 (35.44)	
Mannitol (%)				<0.001
No	2,344 (87.11)	2,109 (88.80)	235 (74.37)	
Yes	347 (12.89)	266 (11.20)	81 (25.63)	
Vasopressors (%)				<0.001
No	2,132 (79.23)	1918 (80.76)	214 (67.72)	
Yes	559 (20.77)	457 (19.24)	102 (32.28)	
Anticoagulants (%)	1,172 (43.55)	1,035 (43.58)	137 (43.35)	0.988
No	1,172 (43.55)	1,035 (43.58)	137 (43.35)	
Yes	1,519 (56.45)	1,340 (56.42)	179 (56.65)	
Antiplatelets (%)				0.026
No	2,123 (78.89)	1858 (78.23)	265 (83.86)	
Yes	568 (21.11)	517 (21.77)	51 (16.14)	
Diuretic (%)				<0.001
No	2,234 (83.02)	2003 (84.34)	231 (73.10)	
Yes	457 (16.98)	372 (15.66)	85 (26.90)	
Thiamine (%)				0.217
No	2,201 (81.79)	1951 (82.15)	250 (79.11)	
Yes	490 (18.21)	424 (17.85)	66 (20.89)	
Vitamin K (%)				<0.001
No	1952 (72.54)	1759 (74.06)	193 (61.08)	
Yes	739 (27.46)	616 (25.94)	123 (38.92)	
Pltinfusion (%)				<0.001
No	2,427 (90.19)	2,170 (91.37)	257 (81.33)	
Yes	264 (9.81)	205 (8.63)	59 (18.67)	
Sedative (%)				<0.001
No	1,240 (46.08)	1,152 (48.51)	88 (27.85)	
Yes	1,451 (53.92)	1,223 (51.49)	228 (72.15)	
wbc (10^9^/L)	10.40 [7.85, 13.70]	10.30 [7.70, 13.50]	12.10 [8.90, 16.60]	<0.001
rbc (1,012/L)	3.81 [3.34, 4.26]	3.83 [3.37, 4.27]	3.56 [3.11, 4.12]	<0.001
Plateletcount (10^9^/L)	194.00 [152.00, 244.00]	195.00 [154.00, 245.00]	178.00 [134.75, 226.50]	<0.001
rdw (%)	13.60 [12.90, 14.60]	13.50 [12.90, 14.50]	14.30 [13.38, 15.50]	<0.001
Sodium (mEq/L)	139.00 [137.00, 142.00]	139.00 [137.00, 142.00]	139.00 [137.00, 142.00]	0.22
Potassium (mEq/L)	4.00 [3.70, 4.40]	4.00 [3.70, 4.40]	4.10 [3.60, 4.50]	0.055
Calciumtotal (mg/dL)	8.50 [8.00, 9.00]	8.50 [8.00, 8.90]	8.60 [8.00, 9.00]	0.335
Glucose (mg/dL)	124.00 [104.00, 153.00]	122.00 [102.00, 148.00]	151.00 [121.00, 187.00]	<0.001
Aniongap (mEq/L)	14.00 [12.00, 16.00]	14.00 [12.00, 16.00]	15.00 [13.00, 18.00]	<0.001
PT (s)	12.70 [11.80, 14.00]	12.70 [11.80, 13.80]	13.55 [12.30, 15.60]	<0.001
INR	1.20 [1.10, 1.30]	1.10 [1.10, 1.30]	1.20 [1.10, 1.40]	<0.001
Ureanitrogen (mg/dL)	15.00 [11.00, 20.00]	14.00 [10.00, 19.00]	19.00 [14.00, 29.00]	<0.001
Creatinine (mg/dL)	0.80 [0.70, 1.10]	0.80 [0.70, 1.00]	1.00 [0.80, 1.40]	<0.001
Age (years)	66.00 [48.00, 81.00]	64.00 [46.00, 80.00]	76.50 [60.75, 88.00]	<0.001
Weight (kg)	75.00 [63.20, 87.20]	75.00 [63.50, 87.30]	72.25 [60.89, 85.05]	0.037
MBP (mmHg)	86.00 [76.00, 97.00]	86.00 [76.00, 97.00]	85.00 [72.75, 96.00]	0.025
R (breaths/min)	18.00 [15.00, 21.00]	18.00 [15.00, 21.00]	19.00 [16.00, 22.00]	0.063
spo2 (%)	99.00 [96.00, 100.00]	99.00 [96.00, 100.00]	100.00 [97.00, 100.00]	<0.001
T (°C)	36.83 [36.56, 37.22]	36.83 [36.56, 37.22]	36.67 [36.39, 37.22]	<0.001
GCS	13.00 [10.00, 14.00]	13.00 [10.00, 14.00]	9.00 [6.00, 12.00]	<0.001

### Feature selection results

Through the intersection of univariate logistic regression (Supplementary Table S1), LASSO regression (Supplementary Figures S1, S2 and Supplementary Table S2), and the Boruta algorithm (Supplementary Figures S3, S4 and Supplementary Table S3), a total of 12 variables were identified as optimal predictors for inclusion in the final models. Specifically, LASSO regression with 10-fold cross-validation was performed to determine the optimal penalty parameter (lambda). The tuning process yielded a lambda.min of 0.005 and a lambda.1se of 0.016. Based on these criteria, 12 variables were retained. The final predictor set included: demographic characteristics (age); neurological status (GCS score); laboratory parameters (anion gap, blood glucose, PT, RDW, WBC, and urea nitrogen); vital signs (temperature); and clinical interventions or acute complications (acidosis, mannitol use, and sedative use). These 12 selected variables were subsequently used as input features for the development and validation of all machine learning models.

### Multimodel integrated analysis for classification

**Figure 2:**
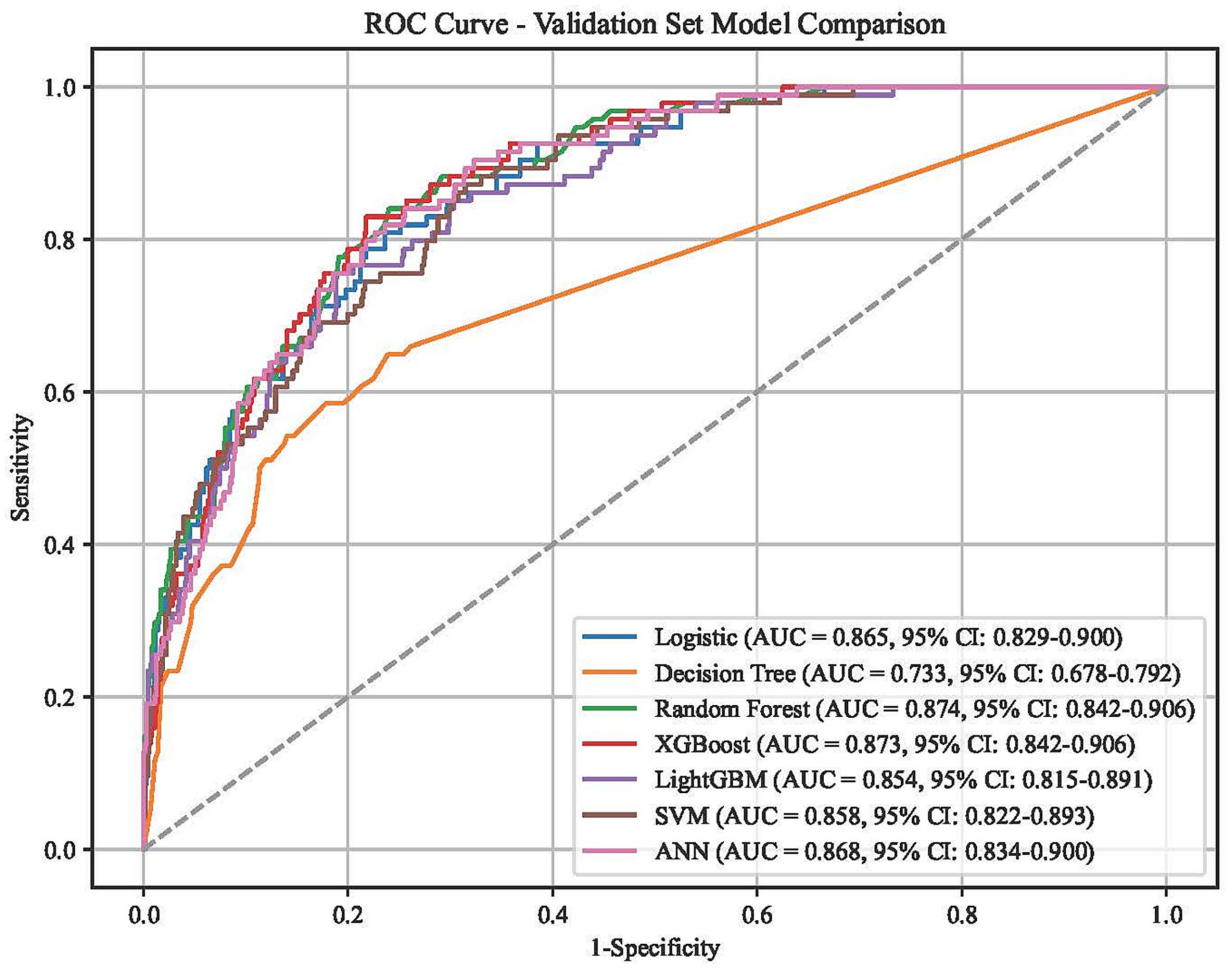
ROC curves of the seven machine learning models in the validation set.

**Figure 3:**
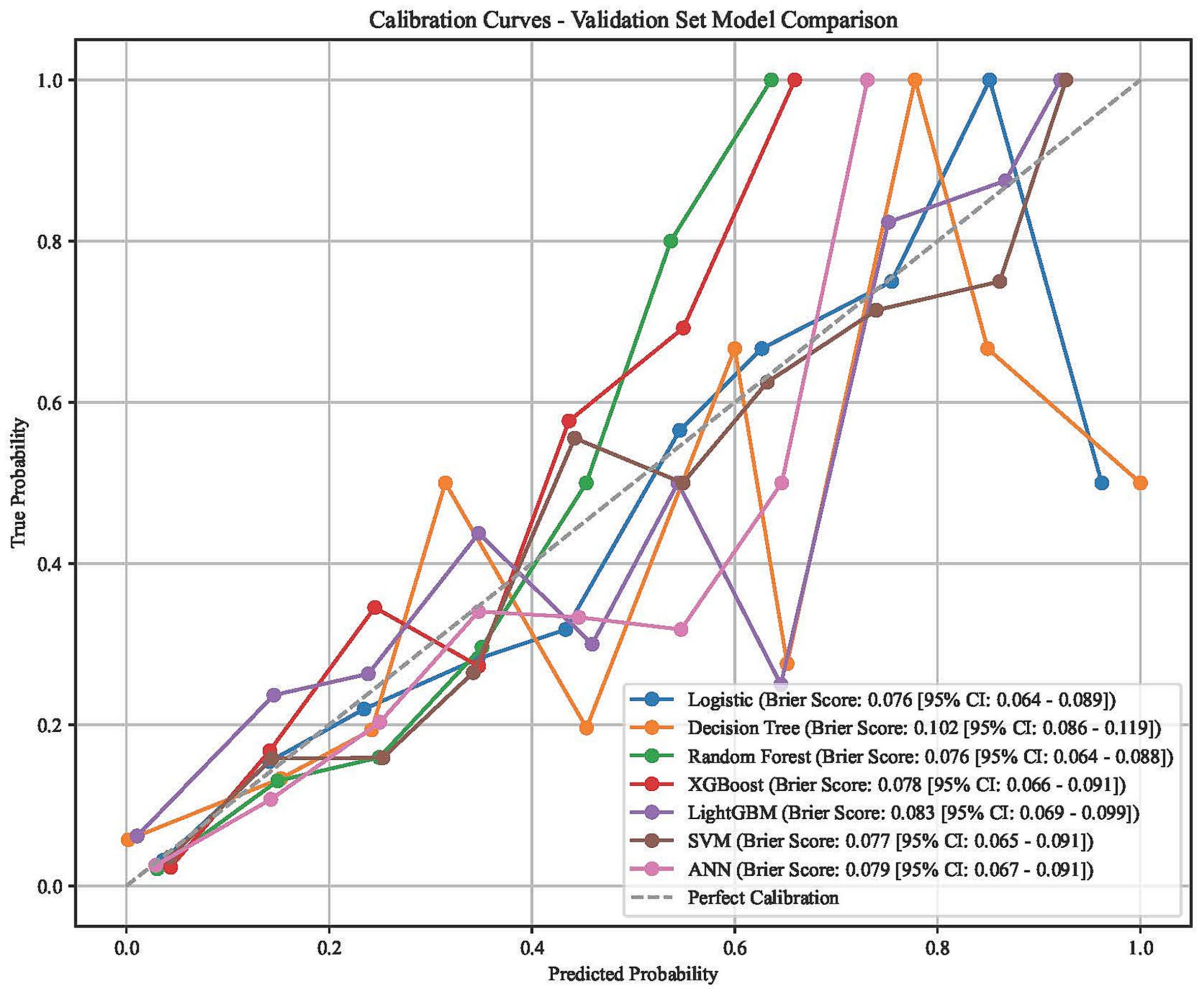
Calibration curves for the seven machine learning models in the validation set.

**Figure 4:**
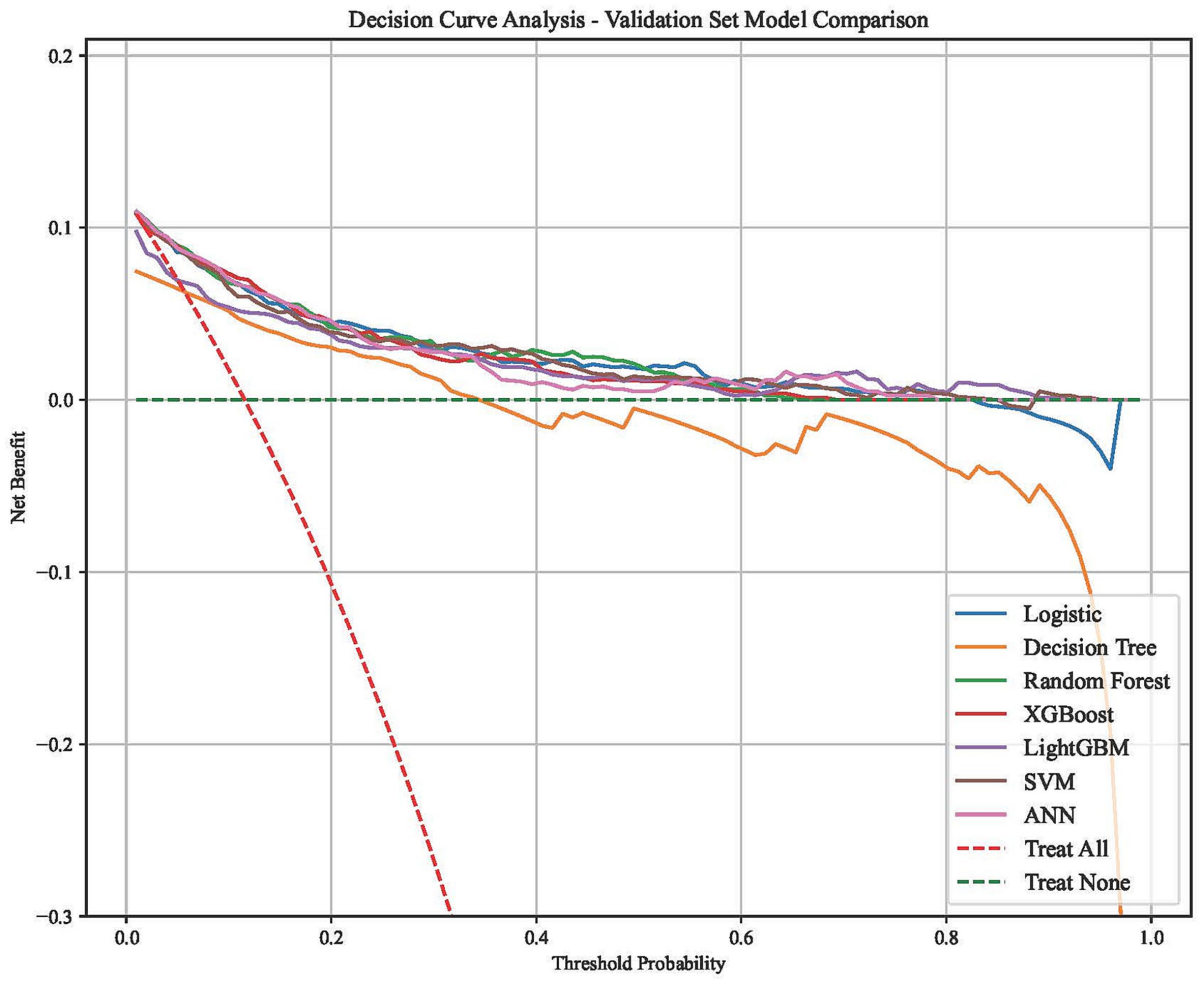
Decision curve analysis (DCA) for the seven machine learning models in the validation set.

Among the seven machine learning models, XGBoost demonstrated superior overall performance across multiple evaluation metrics. In terms of discrimination, the XGBoost model achieved a high AUC of 0.873 (95% CI: 0.842–0.906), which was nearly identical to the Random Forest (AUC: 0.874) and significantly higher than the Decision Tree (AUC: 0.733) (Figure 2). Calibration analysis revealed that the XGBoost model maintained strong agreement between predicted and observed probabilities, yielding a low Brier score of 0.078, outperforming the Decision Tree (0.102) and LightGBM (0.083) (Figure 3). Furthermore, Decision Curve Analysis (DCA) indicated that the XGBoost model provided a consistently high net benefit over the most relevant range of threshold probabilities (Figure 4). Comprehensive metrics summarized in Table 2 confirm that the XGBoost model achieved the best overall balance of predictive power, reaching the highest Accuracy (0.818) and the highest F1 score (0.481) among all models, while maintaining a strong Sensitivity of 0.723 and a Specificity of 0.830. Despite LightGBM showing a higher Precision (0.454) and Specificity (0.917), its significantly lower Sensitivity (0.521) limited its overall utility. Consequently, the XGBoost model was selected as the optimal classifier for subsequent interpretability analysis.

**Table 2:** Performance evaluation of the developed machine learning models in the validation set.

**Model**	**AUC**	**95% CI lower**	**95% CI upper**	**Accuracy**	**Precision**	**Sensitivity**	**Specificity**	**F1 score**	**Kappa**	**Youden’s J**	**PPV**	**NPV**
Logistic	0.865	0.829	0.899	0.811	0.348	0.712	0.824	0.468	0.369	0.537	0.348	0.956
Decision tree	0.733	0.677	0.791	0.784	0.289	0.585	0.810	0.387	0.274	0.395	0.289	0.936
Random forest	0.874	0.842	0.905	0.753	0.300	0.840	0.741	0.442	0.326	0.581	0.300	0.972
XGBoost	0.872	0.842	0.905	0.817	0.359	0.723	0.830	0.480	0.384	0.553	0.359	0.957
LightGBM	0.854	0.815	0.890	0.870	0.453	0.521	0.917	0.485	0.411	0.438	0.453	0.935
SVM	0.858	0.822	0.893	0.795	0.323	0.691	0.808	0.440	0.334	0.500	0.323	0.952
ANN	0.867	0.834	0.899	0.772	0.315	0.808	0.768	0.453	0.343	0.576	0.315	0.968

### Model interpretation

**Figure 5:**
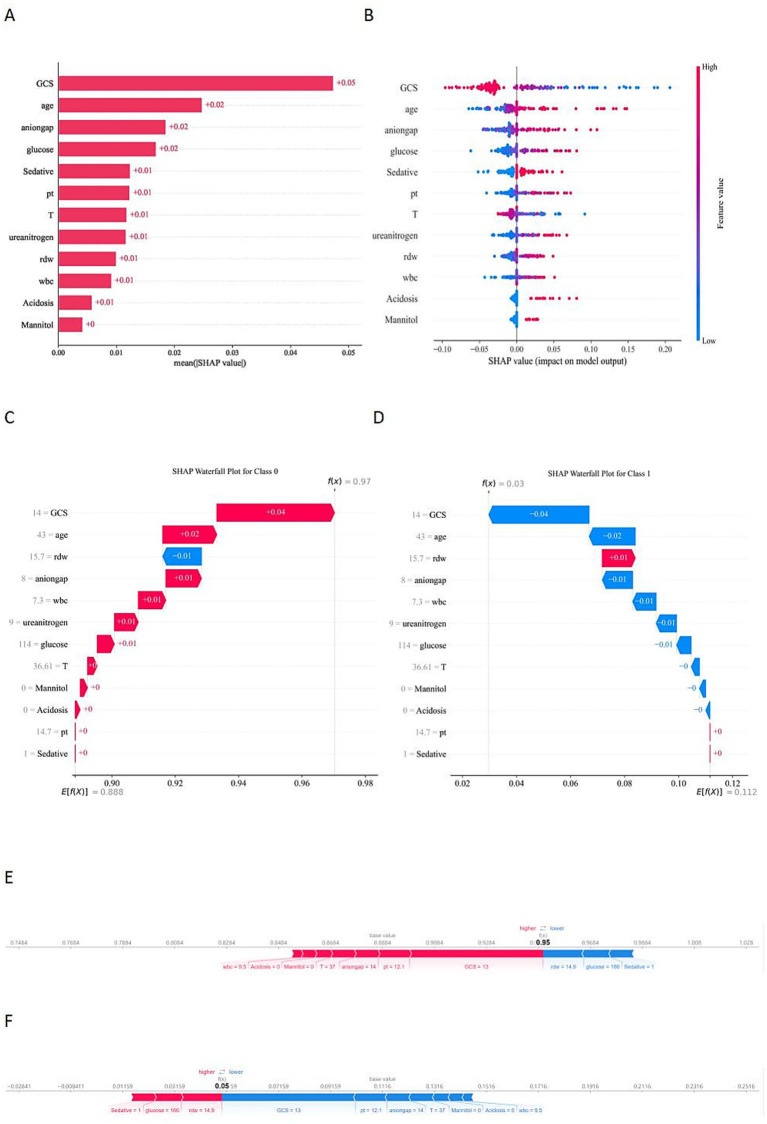
SHAP analysis for model interpretation. **(A)** Summary plot showing the global feature importance ranked by mean absolute SHAP values. **(B)** Beeswarm plot illustrating the distribution of SHAP values for each feature; red represents high feature values, and blue represents low values. **(C,D)** Waterfall plots demonstrating individual feature contributions to the prediction for the first patient in the validation set. **(E,F)** Force plots visualizing the push-and-pull effect of features on the model’s prediction for the third patient in the validation set.

SHAP analysis identified GCS as the most influential predictor in the model, followed by age, anion gap, and glucose (Figure 5A). The beeswarm and dependence plots (Figure 5B and Supplementary Figure S5) further elucidated the directional impact of these features: lower GCS scores (particularly below 8) were strongly associated with an increased risk of the outcome, while advanced age (especially >60 years) and elevated glucose levels (>150 mg/dL) also contributed positively to the risk. Similarly, higher levels of anion gap, prothrombin time (pt), RDW, WBC, and ureanitrogen correlated with higher SHAP values, indicating adverse outcomes. Regarding interventions and clinical conditions, the presence of acidosis, as well as the use of sedatives and mannitol, were indicative of higher risk. Notably, body temperature exhibited a non-linear U-shaped relationship, where deviations from normothermia (<36 °C or >38 °C) were associated with escalated mortality probability.

Individual predictions were visualized using waterfall and force plots (Figures 5C–F). For a representative survivor (Patient 1, Figures 5C,D), the model predicted a high probability of 0.97, primarily driven by a high GCS score (14), younger age (43), and a normal anion gap (8), which outweighed minor negative contributors like an elevated RDW (15.7). In contrast, for Patient 2 (predicted probability: 0.05, Figures 5E,F), although minor factors such as sedative use, elevated glucose (166 mg/dL), and RDW (14.9) pushed towards a higher value, these were completely offset by major negative drivers including a lower GCS score (13), elevated anion gap (14), and prolonged prothrombin time (PT 12.1).

## Discussion

In this study, we analyzed 2,691 ICU patients with traumatic brain injury (TBI) from the MIMIC-IV database, observing an in-hospital mortality rate of 11.7%. By systematically integrating univariate analysis, LASSO regression, and the Boruta algorithm, we identified 12 independent predictors from over 40 candidate variables and successfully developed an interpretable machine learning framework for mortality prediction. Our findings demonstrate that among the seven candidate algorithms, the XGBoost model achieved the most robust performance, yielding a superior AUC of 0.873 and excellent calibration. Notably, the integration of SHAP analysis allowed us to transcend the “black-box” nature of traditional ML, identifying GCS, age, and anion gap as the primary mortality drivers while uncovering a critical non-linear U-shaped relationship between body temperature and the clinical outcome.

Previous studies have robustly demonstrated that the 12 variables identified in this study span multiple dimensions including disease severity, physiological reserve, metabolic homeostasis, inflammatory response, and organ function, all of which are closely associated with outcomes in critically ill patients. Consistent with landmark trials like IMPACT and CRASH, our SHAP analysis identified the Glasgow Coma Scale (GCS) as the strongest independent predictor of TBI mortality, where lower scores—reflecting severe neurological impairment—dictate a higher mortality risk (9, 18). Advanced age, as a non-modifiable prognostic factor, reflects decreased physiological reserve, increased comorbidity burden, and diminished capacity for repair following trauma and secondary injury; prospective cohort studies have confirmed age as a strong predictor of poor functional outcome at 6 months post-TBI (19, 20).

Early hyperglycemia has been reported as a primary predictor of ICU mortality in traumatic brain injury patients (21). Regarding metabolic indicators, hyperglycemia serves not only as a marker of stress response but also directly participates in secondary brain injury pathophysiology by exacerbating cerebral ischemia–reperfusion injury, promoting inflammatory cytokine release, and disrupting blood–brain barrier integrity; admission glucose >200 mg/dL has been independently associated with a 2–3 fold increase in mortality among TBI patients (22, 23). The elevated anion gap and acidosis identified in this study indicate tissue hypoperfusion and anaerobic metabolism leading to lactate accumulation, reflecting severe systemic oxygen supply–demand imbalance; research demonstrates that admission arterial lactate >2.5 mmol/L is an independent predictor of mortality in TBI and correlates with elevated intracranial pressure and reduced brain tissue oxygen tension (24, 25). RDW as a sensitive marker of systemic inflammation and oxidative stress, reflects impaired erythropoiesis and bone marrow dysfunction in response to inflammatory stimuli; in critically ill patients, elevated RDW correlates with increased pro-inflammatory cytokines and has been identified as an independent predictor of poor outcomes following acute brain injury (26, 27). Elevated WBC count indicates SIRS or secondary infection, with excessive systemic inflammatory responses leading to neurotoxic substance release, blood–brain barrier disruption, and neuronal apoptosis (28, 29). Prolonged PT suggests trauma-induced coagulopathy (TIC), which is particularly common in traumatic brain injury; brain tissue is rich in tissue factor, and its release into circulation following injury activates the extrinsic coagulation pathway, while coagulopathy itself exacerbates progressive intracranial hemorrhage and cerebral edema, creating a vicious cycle (30, 31). In neurocritical care, elevated BUN levels typically reflect acute kidney injury (AKI), systemic hypoperfusion, or a severe stress-induced hypercatabolic state following major trauma. Previous studies have consistently demonstrated that early renal dysfunction and elevated BUN are independent risk factors for systemic complications and in-hospital mortality in patients with severe brain injury (32, 33). Notably, temperature demonstrates a U-shaped relationship with outcomes: therapeutic hypothermia (<36 °C) may reduce cerebral metabolic rate but increases risks of infection and coagulopathy, whereas fever (>38 °C) significantly increases cerebral metabolic demand, glutamate release, and free radical generation, aggravating neural injury in the ischemic penumbra; multiple studies have confirmed that post-admission fever is strongly associated with poor outcomes in TBI (34, 35). The association of treatments like mannitol and sedatives with higher mortality in our model reflects confounding by indication. Because these interventions are specifically reserved for critical conditions—such as severe intracranial hypertension or mechanical ventilation—the model utilizes them as surrogate markers of underlying disease severity, rather than causal factors for poor outcomes. Decisions regarding medical management and the withdrawal of care are profoundly influenced by cultural norms, customs, and socioeconomic status. Consequently, prolonged sedation is not a uniform practice across the entire spectrum of traumatic brain injury (TBI); notably, families from disadvantaged socioeconomic backgrounds may contribute to extended sedation periods for their relatives (7, 36, 37).

The XGBoost model developed in this study demonstrated favorable discriminative ability (AUC = 0.873, 95% CI: 0.842–0.906) and calibration (Brier score = 0.078), significantly outperforming the Decision Tree model (AUC = 0.733). While maintaining high specificity (83.0%), the model achieved a robust sensitivity of 72.3%. Decision Curve Analysis confirmed that the model provides consistently high net clinical benefit over the most relevant range of threshold probabilities.

The classic models, such as IMPACT and CRASH (9, 18), have solidified the role of core predictors like age and GCS score. However, their logistic regression algorithms, based on linear assumptions, struggle to capture complex non-linear interactions between physiological parameters and mortality risk. Although Cui et al. (38) recently attempted to introduce machine learning to enhance predictive performance, their study was limited to a small sample (*n* = 230) of patients undergoing decompressive craniectomy. This restriction limits generalizability, and the study failed to address the “black box” issue of model interpretability. Furthermore, while the EPPM model developed by Yang et al. (39) improved accuracy by incorporating the APOE gene and inflammatory factors (IL-8, CRP), the high cost and long turnaround time of these non-routine tests severely restrict their clinical accessibility and utility in acute care settings. Compared to these previous models, our study demonstrates significant methodological and clinical advantages. First, unlike the traditional linear models (9, 18), our XGBoost algorithm effectively captures complex non-linear relationships among clinical variables, thereby achieving robust and superior discrimination in the validation set. Importantly, rather than relying solely on early-phase clinical parameters, the model’s predictive power stems from integrating baseline demographics (age) and admission data (GCS, routine labs) with subsequent ICU complications (sepsis, acidosis) and medical interventions (mannitol, sedatives). Second, regarding clinical utility, unlike Yang et al. (39), who relied on expensive genetic testing or specific biomarkers, the 12 predictors selected in our model are all routine ICU monitoring items. This allows for automated real-time prediction based directly on electronic health records without additional testing costs or waiting times, greatly enhancing clinical accessibility and promotional value. Moreover, in contrast to the study by Cui et al. (38) which was limited to a specific surgical subgroup, our study covers a broad spectrum of TBI patients with varying severity, offering superior generalizability. Addressing the lack of model interpretability in previous machine learning studies, this study innovatively introduces SHAP analysis to crack the “black box” problem. By visually demonstrating individual-level risk drivers through waterfall and force plots, we not only enhance clinician trust in AI decision-making but also provide visual evidence for formulating personalized intervention strategies. Finally, regarding robustness, this study included 2,691 patients based on the MIMIC-IV database, a sample size significantly superior to previous single-center studies (38, 39).

## Limitations

Our study has several limitations. First, regarding generalizability, the retrospective, single-center design (MIMIC-IV) and lack of an external validation cohort introduce selection bias and limit the confirmation of model robustness across diverse settings. Second, the model relies on static variables from the first 24 h of ICU admission, failing to capture the longitudinal evolution of TBI (e.g., fluctuating GCS and serial laboratory trends) essential for identifying delayed deterioration. Third, data constraints precluded the inclusion of granular neuro-monitoring [e.g., ICP, PbtO2, NIRS (40)] and specific neuroimaging parameters (e.g., hemorrhage volume), which may constrain predictive precision. Fourth, treatment variables like mannitol and sedatives act as surrogate markers of extreme disease severity due to confounding by indication, rather than causal risk factors. Finally, despite satisfactory sensitivity (72.3%) and high negative predictive value (95.8%), precision (36.0%) and F1-score (0.481) remain modest. Attempts to mitigate this class imbalance using SMOTE yielded limited improvements. While prioritizing sensitivity inevitably generates more false-positive alerts, this trade-off is clinically justifiable for an early screening tool: it safely ‘rules out’ low-risk patients to minimize missed deteriorations, allowing clinicians to dynamically calibrate thresholds to balance sensitivity against alarm fatigue.

## Conclusion

In conclusion, we successfully developed and validated an interpretable machine learning model for predicting in-hospital mortality among ICU patients with traumatic brain injury. Through rigorous multi-algorithm comparison, the XGBoost classifier emerged as the superior model, demonstrating robust discriminative capacity, excellent calibration, and high sensitivity. With its strong negative predictive value, the model serves as a reliable early risk stratification tool to safely ‘rule out’ low-risk patients. Furthermore, by integrating SHAP-based explainability, this study provides both population-level insights into key risk drivers and individualized risk attribution, effectively bridging the gap between predictive accuracy and clinical interpretability. Future integration of longitudinal data and prospective multicenter validation are warranted to confirm these findings before widespread clinical deployment.

## Data Availability

The original contributions presented in the study are included in the article/Supplementary material, further inquiries can be directed to the corresponding author.
