# Supplementary material for: Interpretable machine learning for in-hospital mortality prediction in ICU patients with traumatic brain injury
Source: Front Neurol. 2026 Apr 23;17:1815307. doi: 10.3389/fneur.2026.1815307 (PMC13149133; doi:10.3389/fneur.2026.1815307)
Supplement: Supplementary file 1 [file Data_Sheet_1.ZIP › Supplement figure legend/Model assessment without the use of mannitol and sedatives.docx]

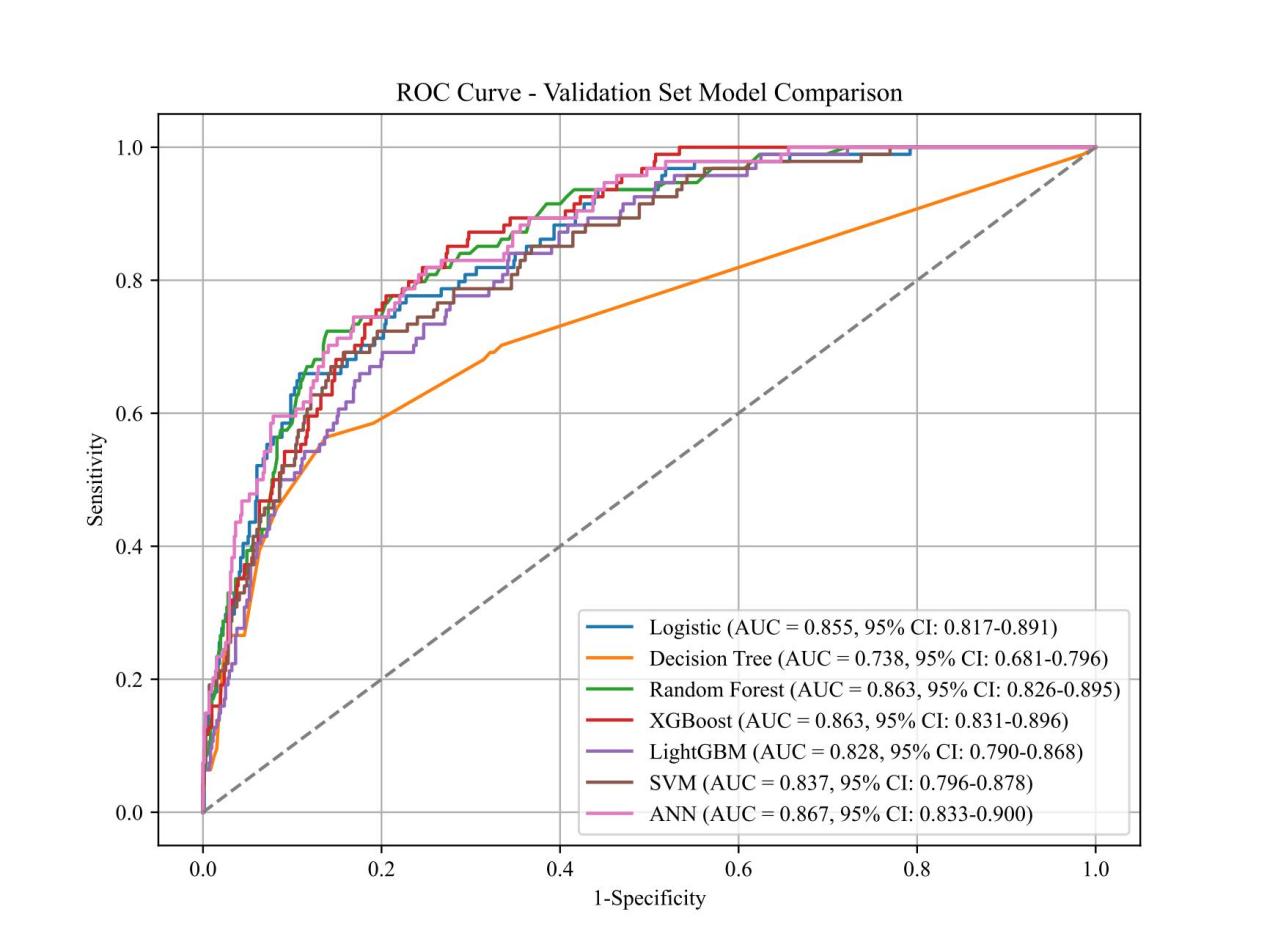


Fig.1 ROC curves of the seven machine learning models in the validation set.


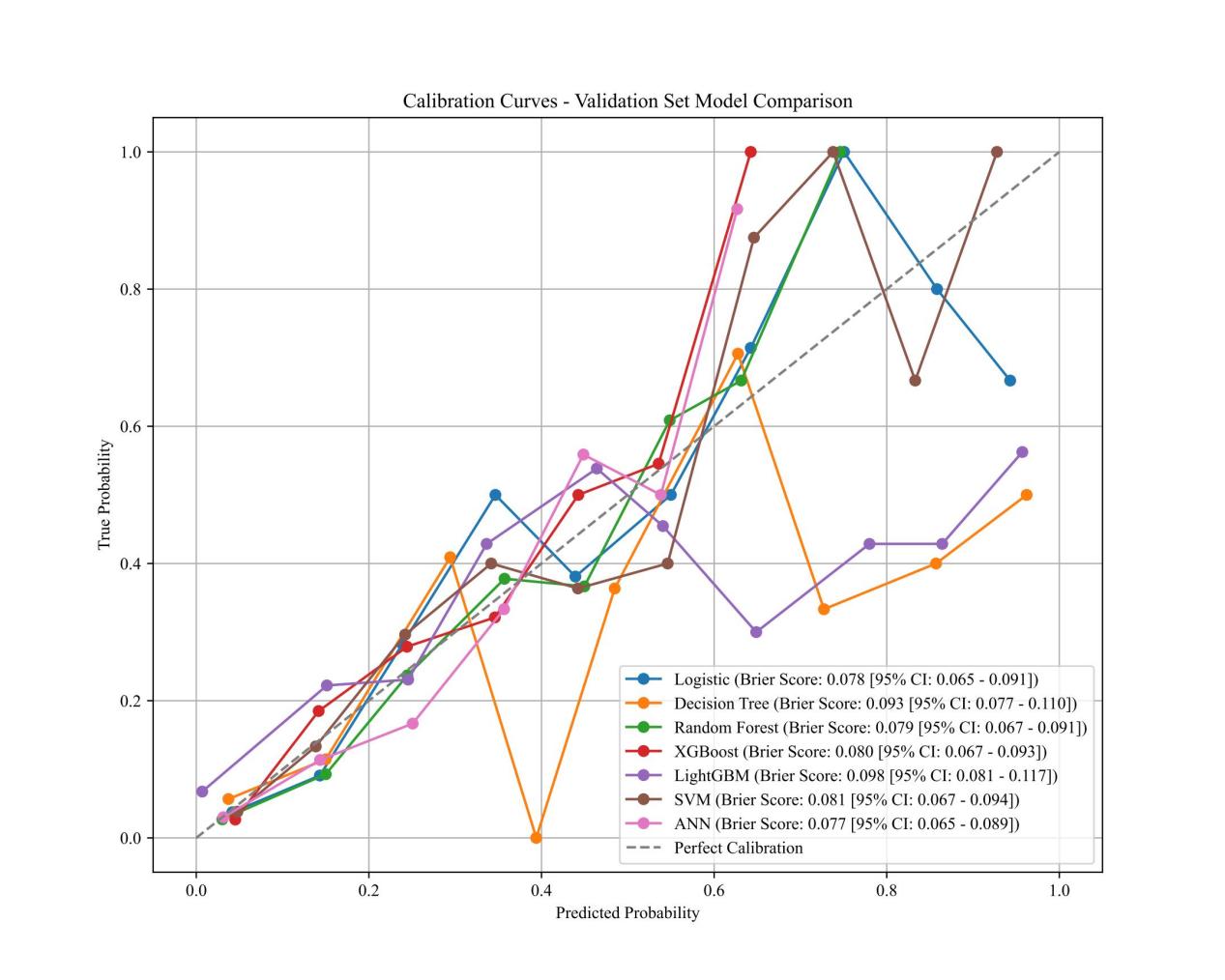


Fig.2 Calibration curves for the seven machine learning models in the validation set.


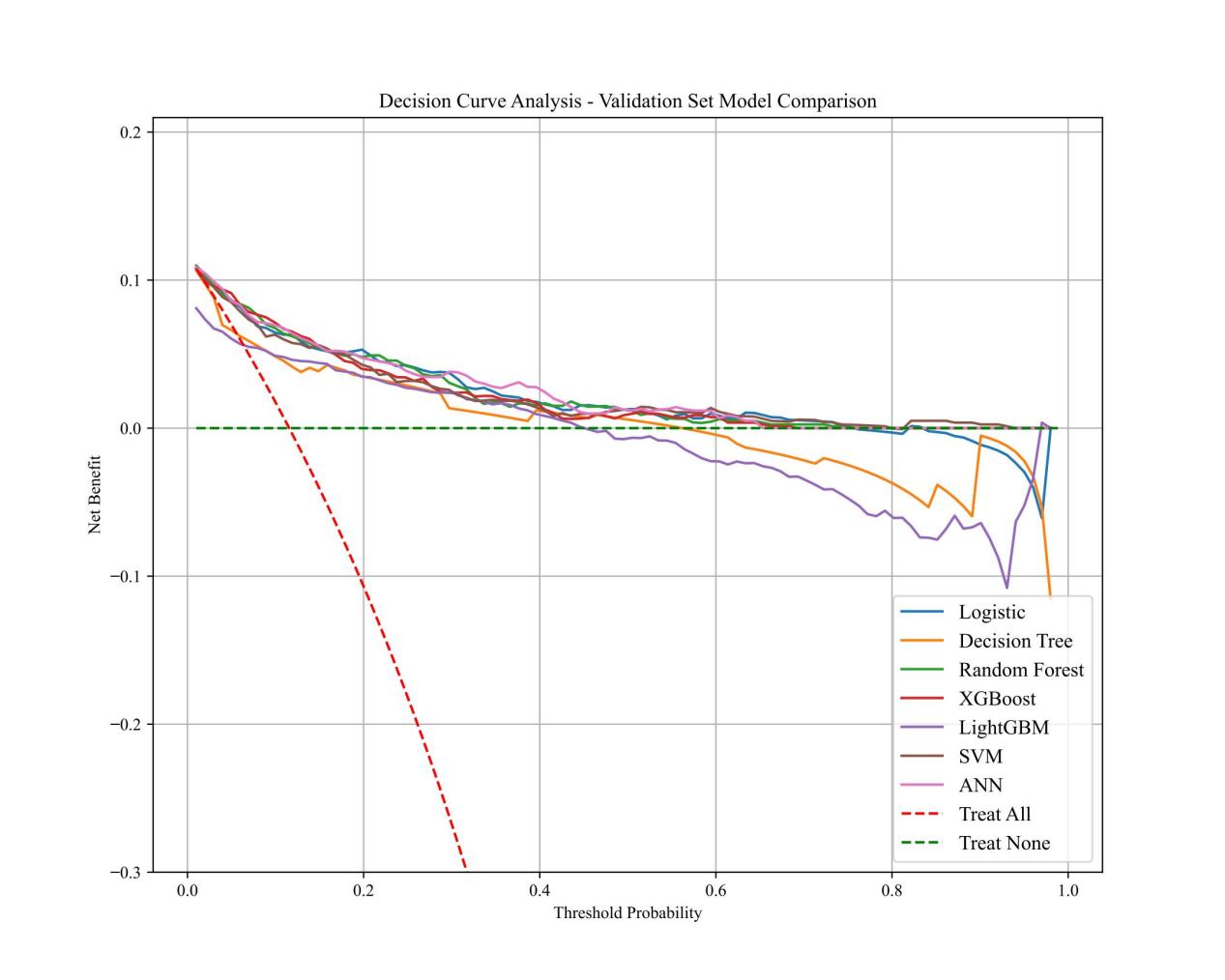


Fig.3 Decision curve analysis (DCA) for the seven machine learning models in the validation set.

**Table 1** Performance evaluation of the developed machine learning models in the validation set.

| Model | AUC | 95% CI Lower | 95% CI Upper | Accuracy | Precision | Sensitivity | Specificity | F1 Score | Kappa | Youden's J | PPV | NPV |  |
| --- | --- | --- | --- | --- | --- | --- | --- | --- | --- | --- | --- | --- | --- |
| Logistic | 0.8545900071718863 | 0.8170732723438485 | 0.890594641648726 | 0.8089330024813896 | 0.34210526315789475 | 0.6914893617021277 | 0.824438202247191 | 0.45774647887323944 | 0.3574830213682292 | 0.5159275639493188 | 0.34210526315789475 | 0.952922077922078 |  |
| Decision Tree | 0.737934795601243 | 0.6807901481637526 | 0.7964162229120071 | 0.78287841191067 | 0.2879581151832461 | 0.5851063829787234 | 0.8089887640449438 | 0.3859649122807018 | 0.2721953333814924 | 0.3940951470236671 | 0.2879581151832461 | 0.9365853658536586 |  |
| Random Forest | 0.8628376763088692 | 0.8259913893189226 | 0.8952693730465272 | 0.7543424317617866 | 0.296875 | 0.8085106382978723 | 0.7471910112359551 | 0.4342857142857142 | 0.31791838339630385 | 0.5557016495338274 | 0.296875 | 0.9672727272727273 |  |
| XGBoost | 0.8634353334927086 | 0.8308653123283983 | 0.8957441930649438 | 0.8089330024813896 | 0.34536082474226804 | 0.7127659574468085 | 0.8216292134831461 | 0.46527777777777773 | 0.36560085047225743 | 0.5343951709299546 | 0.34536082474226804 | 0.9558823529411765 |  |
| LightGBM | 0.8280988524982071 | 0.7901853074349656 | 0.8676995085727338 | 0.8610421836228288 | 0.41964285714285715 | 0.5 | 0.9087078651685393 | 0.45631067961165045 | 0.37734859980686997 | 0.4087078651685392 | 0.41964285714285715 | 0.9322766570605188 |  |
| SVM | 0.8367947645230697 | 0.7957337063298688 | 0.8775080885493852 | 0.825062034739454 | 0.3672316384180791 | 0.6914893617021277 | 0.8426966292134831 | 0.4797047970479705 | 0.38619497704563865 | 0.5341859909156108 | 0.3672316384180791 | 0.9538950715421304 |  |
| ANN | 0.8673499880468563 | 0.8325038718297235 | 0.9003796915088206 | 0.7816377171215881 | 0.3185840707964602 | 0.7659574468085106 | 0.7837078651685393 | 0.45 | 0.34152772104422735 | 0.54966531197705 | 0.3185840707964602 | 0.9620689655172414 |  |
