# Supplementary material for: Interpretable machine learning for in-hospital mortality prediction in ICU patients with traumatic brain injury
Source: Front Neurol. 2026 Apr 23;17:1815307. doi: 10.3389/fneur.2026.1815307 (PMC13149133; doi:10.3389/fneur.2026.1815307)
Supplement: Supplementary file 1 [file Data_Sheet_1.ZIP › Supplement figure legend/Supplement figure legend.docx]

**Supplement Figure S1**

Selection of optimal tuning parameter (λ) in the LASSO model via 10-fold cross-validation.

**Supplement Figure S2**

LASSO coefficient profiles of the clinical potential predictors.

**Supplement Figure S3**

Iterative feature selection process using the Boruta algorithm.

**Supplement Figure S4**

Variable importance ranking for predicting in-hospital mortality in TBI patients based on the Boruta algorithm.

**Supplement Figure S5**

SHAP dependence plots illustrating the non-linear relationships between key predictors and the risk of in-hospital mortality.
