# Supplementary material for: Interpretable machine learning for in-hospital mortality prediction in ICU patients with traumatic brain injury
Source: Front Neurol. 2026 Apr 23;17:1815307. doi: 10.3389/fneur.2026.1815307 (PMC13149133; doi:10.3389/fneur.2026.1815307)
Supplement: Supplementary file 1 [file Data_Sheet_1.ZIP › Supplement figure legend/Table S1.docx]

**Univariate Logistic Regression Analysis of Potential Predictors for In-hospital Mortality in the Validation Set.**

| **Varialbes** | **P值** |
| --- | --- |
| AKI | <0.001 |
| CHD | 0.33 |
| Epilepsy | 0.711 |
| hypertension | 0.659 |
| diabetes | <0.001 |
| HF | <0.001 |
| cancer | 0.184 |
| chronic_renal | <0.001 |
| stroke | 0.006 |
| pneumonia | 0.002 |
| sepsis3 | <0.001 |
| Acidosis | <0.001 |
| delirium | 1 |
| gender | 0.002 |
| marital_status | 0.008 |
| Mechanical_ventilation | 0.002 |
| Neurosurgical_surgery | 0.06 |
| Tracheostomy | 0.193 |
| betablocker | 0.121 |
| Mannitol | <0.001 |
| Vasopressors | <0.001 |
| Anticoagulants | 0.988 |
| Antiplatelets | 0.026 |
| Diuretic | <0.001 |
| Thiamine | 0.217 |
| Vitamin_K | <0.001 |
| pltinfusion | <0.001 |
| Sedative | <0.001 |
| wbc | <0.001 |
| rbc | <0.001 |
| plateletcount | <0.001 |
| rdw | <0.001 |
| sodium | 0.187 |
| potassium | 0.038 |
| calciumtotal | 0.286 |
| glucose | <0.001 |
| aniongap | <0.001 |
| pt | <0.001 |
| inr | <0.001 |
| ureanitrogen | <0.001 |
| creatinine | <0.001 |
| age | <0.001 |
| weight | 0.037 |
| MBP | 0.02 |
| R | 0.057 |
| spo2 | <0.001 |
| T | <0.001 |
| GCS | <0.001 |

| **Varialbes** | **P** |
| --- | --- |
| AKI (%) | <0.001 |
| CHD (%) | 0.33 |
| Epilepsy (%) | 0.711 |
| hypertension (%) | 0.659 |
| diabetes (%) | <0.001 |
| HF (%) | <0.001 |
| cancer (%) | 0.184 |
| chronic_renal (%) | <0.001 |
| stroke (%) | 0.006 |
| pneumonia (%) | 0.002 |
| sepsis3 (%) | <0.001 |
| Acidosis (%) | <0.001 |
| delirium (%) | 1 |
| gender (%) | 0.002 |
| marital_status_M_D_O (%) | 0.008 |
| Mechanical_ventilation (%) | 0.002 |
| Neurosurgical_surgery (%) | 0.06 |
| Tracheostomy (%) | 0.193 |
| betablocker (%) | 0.121 |
| Mannitol (%) | <0.001 |
| Vasopressors (%) | <0.001 |
| Anticoagulants (%) | 0.988 |
| Antiplatelets (%) | 0.026 |
| Diuretic (%) | <0.001 |
| Thiamine (%) | 0.217 |
| Vitamin_K (%) | <0.001 |
| pltinfusion (%) | <0.001 |
| Sedative (%) | <0.001 |
| wbc (median [IQR]) | <0.001 |
| rbc (median [IQR]) | <0.001 |
| plateletcount (median [IQR]) | <0.001 |
| rdw (median [IQR]) | <0.001 |
| sodium (median [IQR]) | 0.187 |
| potassium (median [IQR]) | 0.038 |
| calciumtotal (median [IQR]) | 0.286 |
| glucose (median [IQR]) | <0.001 |
| aniongap (median [IQR]) | <0.001 |
| pt (median [IQR]) | <0.001 |
| inr (median [IQR]) | <0.001 |
| ureanitrogen (median [IQR]) | <0.001 |
| creatinine (median [IQR]) | <0.001 |
| age (median [IQR]) | <0.001 |
| weight (median [IQR]) | 0.037 |
| MBP (median [IQR]) | 0.02 |
| R (median [IQR]) | 0.057 |
| spo2 (median [IQR]) | <0.001 |
| T (median [IQR]) | <0.001 |
| GCS (median [IQR]) | <0.001 |
