# Supplementary material for: Interpretable machine learning for in-hospital mortality prediction in ICU patients with traumatic brain injury
Source: Front Neurol. 2026 Apr 23;17:1815307. doi: 10.3389/fneur.2026.1815307 (PMC13149133; doi:10.3389/fneur.2026.1815307)
Supplement: Supplementary file 1 [file Data_Sheet_1.ZIP › Supplement figure legend/Table S2.docx]

**Selection of clinical features and their corresponding coefficients using the LASSO regression model.**

| **Variables** | **s0** |
| --- | --- |
| (Intercept) | -3.770741459 |
| AKI | 0.296294849418058 |
| CHD | 0 |
| Epilepsy | 0 |
| hypertension | 0 |
| diabetes | 0 |
| HF | 0 |
| cancer | 0 |
| chronic_renal | 0 |
| stroke | 0 |
| pneumonia | 0 |
| sepsis3 | 0 |
| Acidosis | 0.641539547896302 |
| delirium | -0.060186899 |
| gender | 0 |
| marital_status_M_D_O | 0 |
| Mechanical_ventilation | 0 |
| Neurosurgical_surgery | 0 |
| Tracheostomy | 0 |
| betablocker | 0 |
| Mannitol | 0.488501529568533 |
| Vasopressors | 0 |
| Anticoagulants | 0 |
| Antiplatelets | -0.101781474 |
| Diuretic | 0 |
| Thiamine | 0 |
| Vitamin_K | 0 |
| pltinfusion | 0.0874309056509737 |
| Sedative | 0.335248361327623 |
| wbc | 1.10867928829298 |
| rbc | 0 |
| plateletcount | 0 |
| rdw | 0.605542946760548 |
| sodium | 0 |
| potassium | 0 |
| calciumtotal | 0 |
| glucose | 2.01848887574354 |
| aniongap | 2.41983496643015 |
| pt | 1.57525544811679 |
| inr | 0 |
| ureanitrogen | 1.18592401621486 |
| creatinine | 0 |
| age | 1.39592904285402 |
| weight | 0 |
| MBP | 0 |
| R | 0 |
| spo2 | 0 |
| T | -1.906611797 |
| GCS | -2.058278977 |
