# Supplementary material for: Interpretable machine learning for in-hospital mortality prediction in ICU patients with traumatic brain injury
Source: Front Neurol. 2026 Apr 23;17:1815307. doi: 10.3389/fneur.2026.1815307 (PMC13149133; doi:10.3389/fneur.2026.1815307)
Supplement: Supplementary file 1 [file Data_Sheet_1.ZIP › Supplement figure legend/Table S3.docx]

**Feature selection using the Boruta algorithm for predicting in-hospital mortality in patients with TBI.**

| **Variables** |
| --- |
| chronic_renal |
| pneumonia |
| sepsis3 |
| Acidosis |
| delirium |
| Mechanical_ventilation |
| Tracheostomy |
| Mannitol |
| Vasopressors |
| Anticoagulants |
| Diuretic |
| Vitamin_K |
| Sedative |
| wbc |
| rbc |
| rdw |
| sodium |
| potassium |
| calciumtotal |
| glucose |
| aniongap |
| pt |
| inr |
| ureanitrogen |
| creatinine |
| age |
| weight |
| spo2 |
| T |
| GCS |
