# Supplementary figures and images for: Interpretable machine learning for in-hospital mortality prediction in ICU patients with traumatic brain injury
Source: Front Neurol. 2026 Apr 23;17:1815307. doi: 10.3389/fneur.2026.1815307 (PMC13149133; doi:10.3389/fneur.2026.1815307)

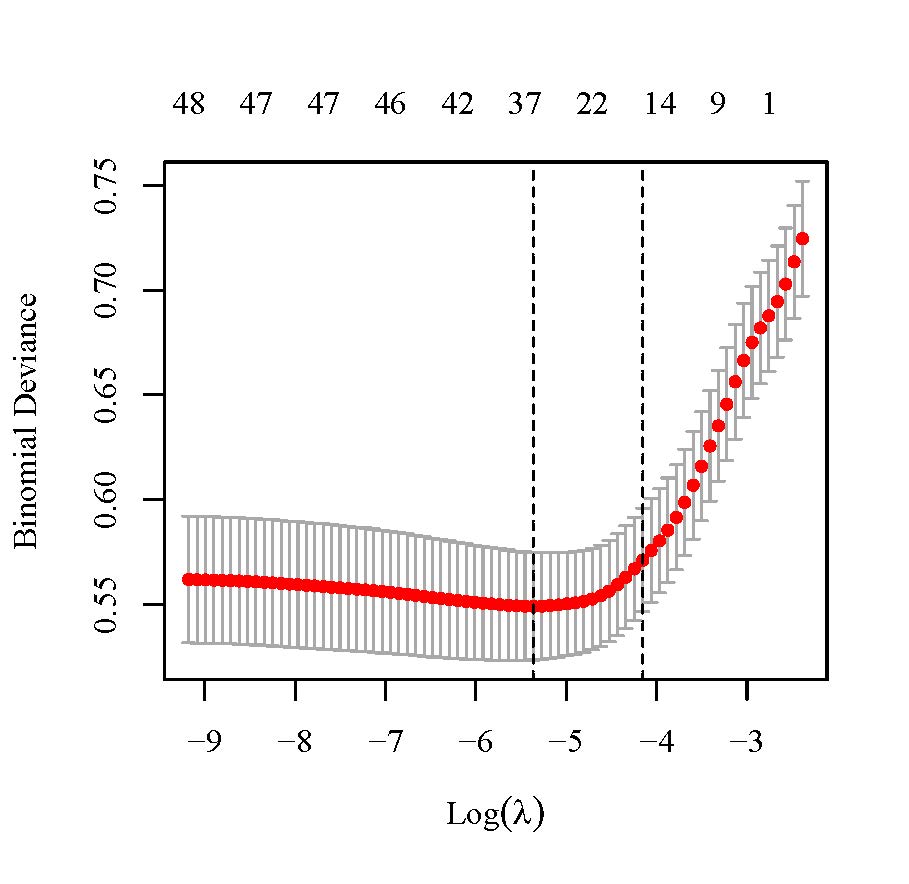

Supplement: Supplementary file 1 [file Data_Sheet_1.ZIP › Supplement figure legend/Figure S1.jpg]

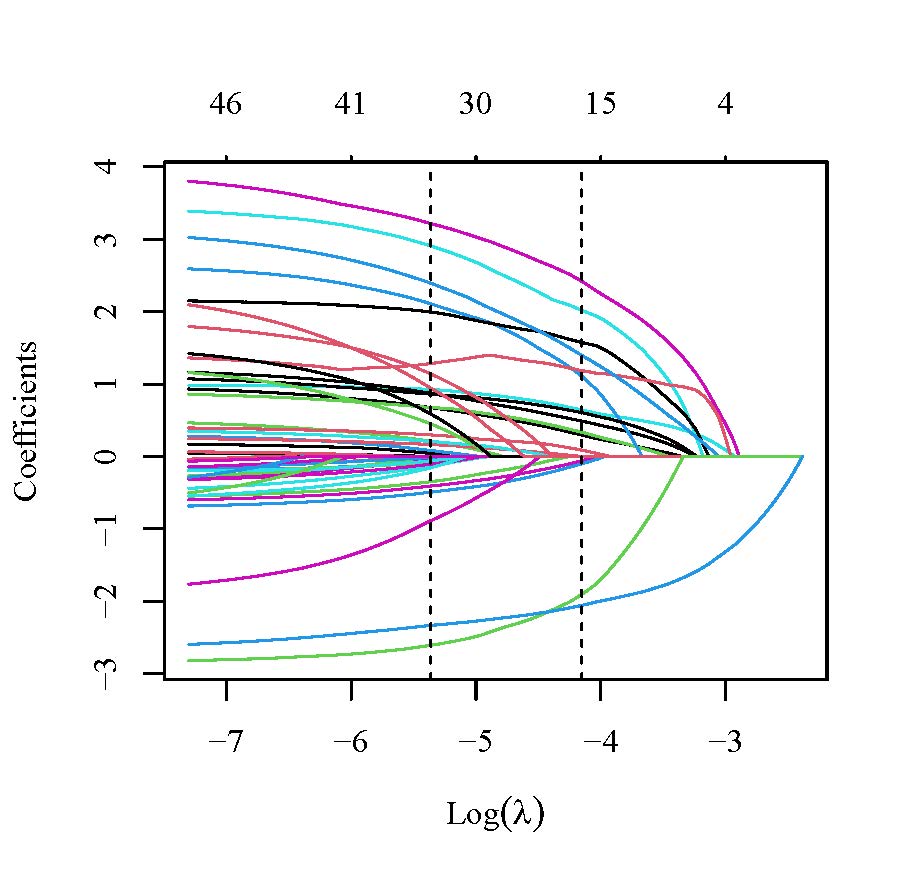

Supplement: Supplementary file 1 [file Data_Sheet_1.ZIP › Supplement figure legend/Figure S2.jpg]

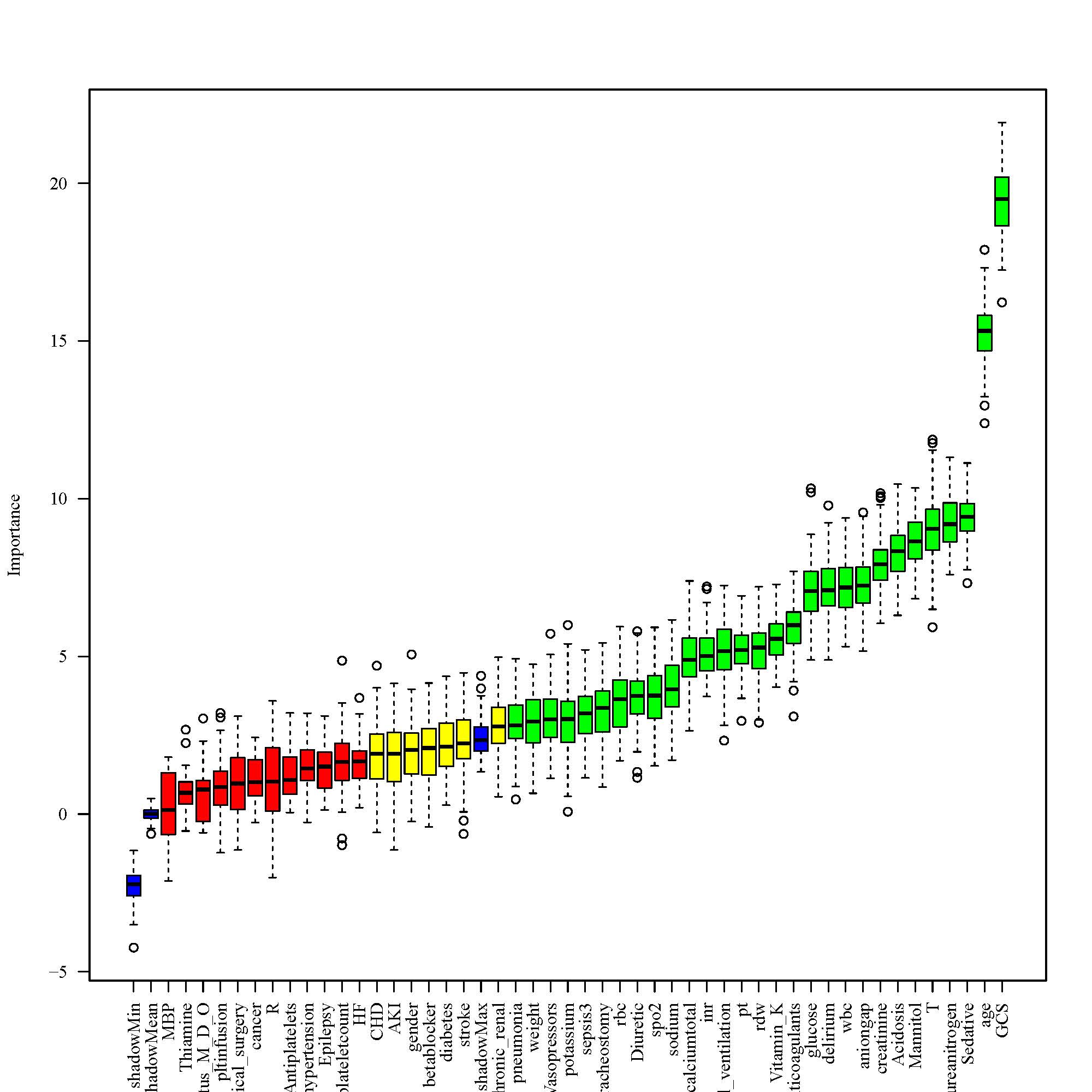

Supplement: Supplementary file 1 [file Data_Sheet_1.ZIP › Supplement figure legend/Figure S3.jpg]

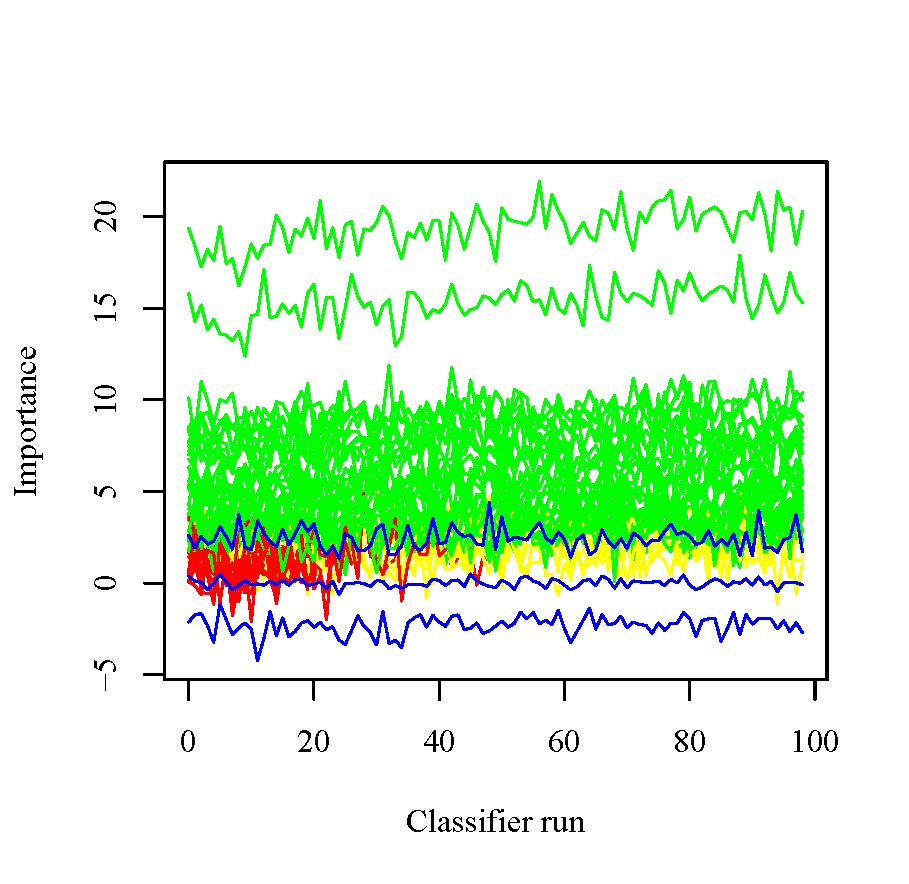

Supplement: Supplementary file 1 [file Data_Sheet_1.ZIP › Supplement figure legend/Figure S4.jpg]

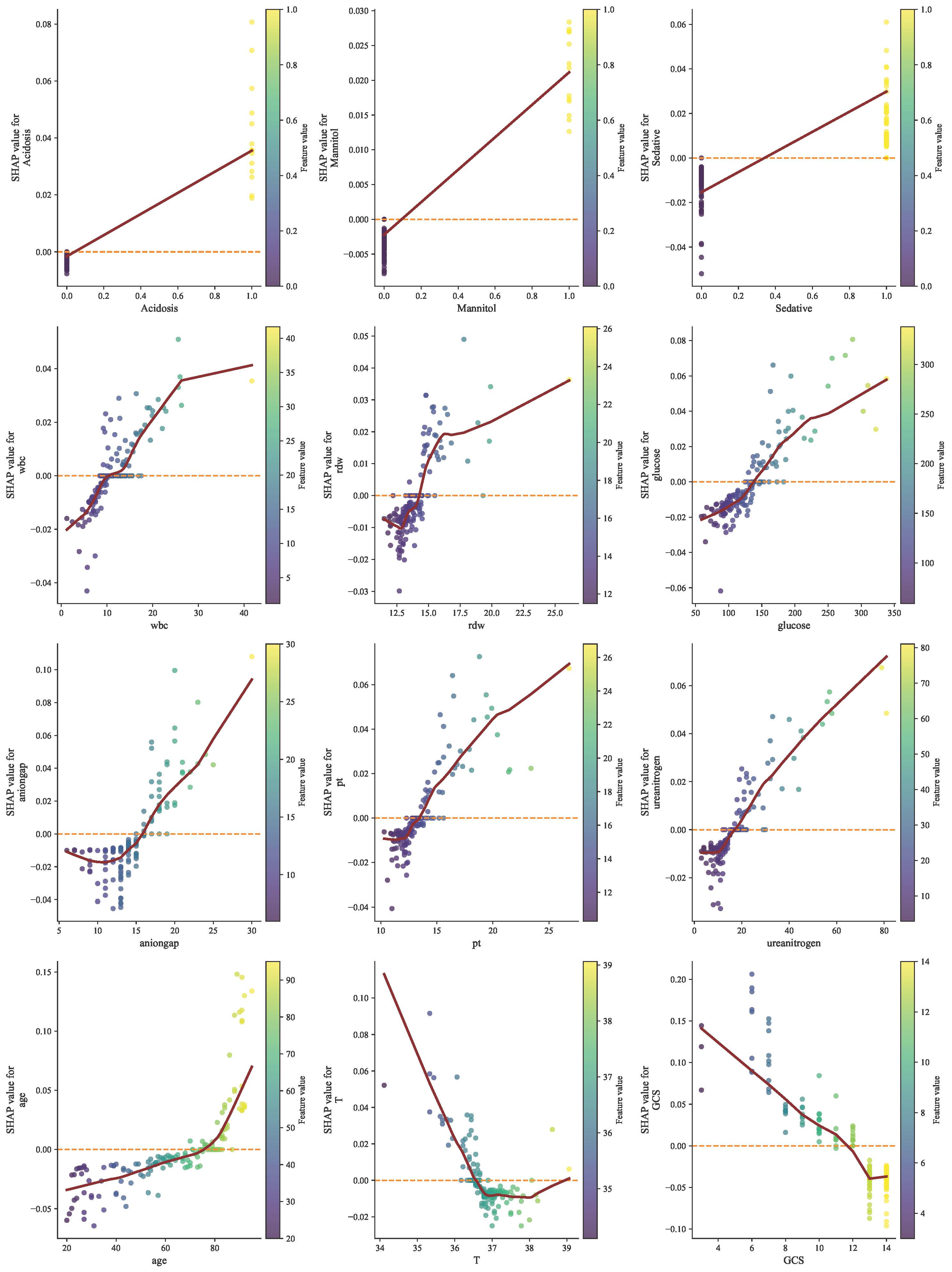

Supplement: Supplementary file 1 [file Data_Sheet_1.ZIP › Supplement figure legend/Figure S5.jpg]
